# Development, implementation, and evaluation of Teach Back curriculum for community health workers

**DOI:** 10.3389/fmed.2022.918686

**Published:** 2022-11-03

**Authors:** Jennifer Holcomb, Gayla M. Ferguson, Logan Thornton, Linda Highfield

**Affiliations:** ^1^Department of Management, Policy and Community Health, The University of Texas Health Science Center at Houston (UTHealth) School of Public Health, Houston, TX, United States; ^2^Division of Population Health and Evidence-Based Practice, Healthcare Transformation Initiatives, The University of Texas Health Science Center at Houston (UTHealth) John P. and Kathrine G. McGovern Medical School, Houston, TX, United States; ^3^Department of Epidemiology, Human Genetics and Environmental Sciences, The University of Texas Health Science Center at Houston (UTHealth) School of Public Health, Houston, TX, United States; ^4^Department of Internal Medicine, The University of Texas Health Science Center at Houston (UTHealth) John P. and Kathrine G. McGovern Medical School, Houston, TX, United States

**Keywords:** Teach Back, community health worker, training, health literacy, curriculum development

## Abstract

Teach Back is a commonly used communication method to improve patient understanding and retention of health information. The method has been shown to be effective in improving patient and healthcare system outcomes, including patient health literacy and hospital readmissions. Community health workers (CHWs) are frontline healthcare workers who can help address patient health and social needs associated with hospital readmissions. However, a gap exists in Teach Back curricula and training methods reflecting the scope of work for CHWs. The objective of this training was to provide CHWs with didactic information and skill building practice curriculum focused on the integration of Teach Back into clinical patient interactions, care coordination, and follow-up support. A multidisciplinary team of academic and clinical partners at a large academic health university developed, implemented, and evaluated a 3-week pilot Teach Back training with CHWs through a quality improvement approach. The CHWs reported overall satisfaction with the training and instructors. The academic clinical partnership allowed the training to be tailored to the daily clinical workflow as reflected in the CHWs agreement that the training was relevant and practical. With the repeated exposure to Teach Back each week, the CHWs also reported an increase in confidence and conviction in using Teach Back. Additional implementation and evaluation of the training curriculum for CHWs is needed to gain further insights into Teach Back and training best practices and translation into practice.

## Introduction

Reducing avoidable readmissions is a national priority in the United States (1, 2). Readmissions are costly to the healthcare system and there is a growing recognition of the role patient social needs play in hospital readmissions (3–5). Social and environmental factors including health literacy, low socioeconomic status, and neighborhood disadvantage (defined by income, employment, education, and housing status) play a significant role in hospital readmissions, particularly in communities who are underserved (6). From the healthcare service delivery vantage point, factors such as access to primary care, hospital discharge planning, and transitions of care coordination are associated with hospital readmissions (7). A growing body of literature suggests reducing unnecessary hospitalization depends on tailoring interventions to accurately identify risk factors in post-discharge care (7). Successful interventions that target patients with high needs and high cost of care share several characteristics. Characteristics include embedded transitions of care coordinators in the ambulatory setting; involvement with social and community services to address patient needs; and effectively communicating care regimens to patients and caregivers (8–10). Scheduling transportation for timely follow-up appointments and filling health literacy gaps can lower readmission rates and facilitate continuity of care (8, 11, 12). Multidisciplinary clinical care teams in ambulatory settings can link patients to supportive services and ensure necessary short- and long-term follow-up. The team-based care approach is a foundational element to transforming clinical care (13, 14).

As an extension of the multidisciplinary care team, community health workers (CHWs) can address some social risk factors like lack of transportation to an appointment that are associated with hospital readmissions (14, 15). CHWs are frontline healthcare workers and cultural members of the populations they serve. CHWs have the ability to connect with patients in a manner that allows patients to feel comfortable opening up about the assistance needed and trust the CHWs to provide it (16, 17). Kwan et al. (17) found CHWs were able to help frequent emergency department users meet 43% of their objectives through linkage to community resources and navigation. Ramos et al. (18) recommended the development of a well-designed and validated program for CHWs working as patient navigators be made a national priority. Public health and, more recently, healthcare institutions have recognized the valuable support CHWs can provide to an overtaxed healthcare system to address the chronic health conditions, medical compliance, and health-related social needs of patients (18–20).

Clear communication is vital to ensuring a patient’s understanding and retention of medical or health information. Reasons why a patient might lack understanding or retention of information include lack of culturally appropriate communication, low health literacy, use of medical jargon, an overestimate of a provider’s own ability to communicate, or lack of time during a visit to communicate information, lack of patient involvement (21–23). Despite the need for more evidence on the appropriate level of patient health literacy and involvement needed in medical decision-making, the Teach Back methodology is a promising communication method to improve patient understanding, retention, and use of information (21, 24, 25). Teach Back uses allows healthcare providers to gauge a patient’s understanding by prompting patients to rephrase in their own words what they have heard and understood (26). Published curriculum and training methods for Teach Back have varied from standard didactic instruction sessions to role playing, demonstration videos, and role modeling (23, 27–30). Kornburger et al. (27) found after a multimodal training including videos, handouts, and guided practice had a more than 40% increase in self-reported use of Teach Back among nurses. Ninety six percent (96%) of nurses in a cardiac unit reported continued use of Teach Back after receiving a demonstration of Teach Back principles followed by guided practice (28). Morony et al. (29) found a 2-h training including role playing, handouts, demonstration videos, and peer learning were effective Teach Back training methods. However, Anderson et al. (30) did not see lasting mastery of proper Teach Back after a 4-h training class and had to add a 2-h refresher courses 5 months after the initial 4-h training reiterating the same points. More examination is needed to determine the necessary dosage to produce effective implementation and translation of Teach Back into practice (23, 30, 31). There is abundant evidence to the significance of use of Teach Back (23). However, current curricula and training revolves around clinical staff such as physicians and nurses and might not reflect CHW scope of work (23, 30–34).

**FIGURE 1:**
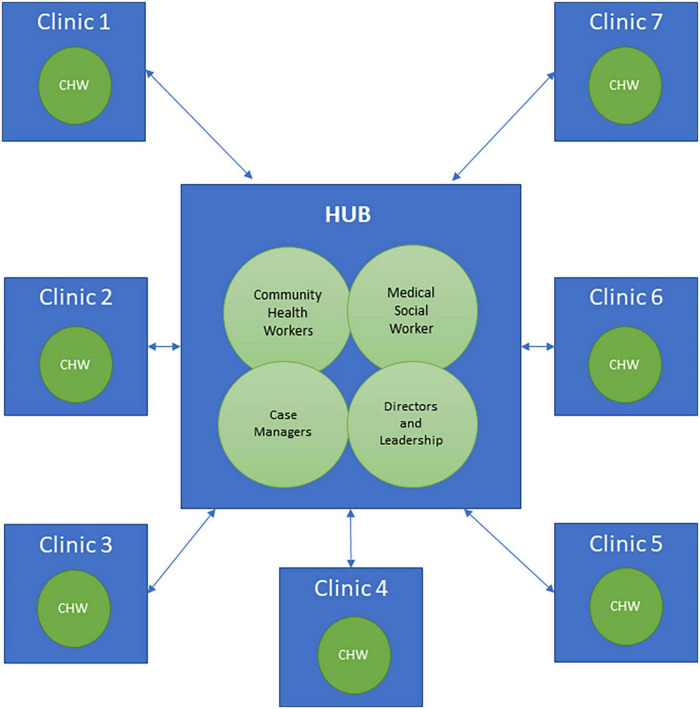
Centralized hub’s relationship with clinics and staff in the academic practice plan.

A project focused on reducing hospital readmissions was established between multidisciplinary academic and clinical partners at a large academic health university. One component of the project was an evidence-based intervention to address readmission. The academic partner had expertise in intervention development and community-based trainings with CHWs providing care to communities that are underserved. The clinical partner is a healthcare transformation initiatives department within the clinical practice of the university’s medical school. The department supports clinical effectiveness and quality and practice improvement of an ambulatory practice plan. Situated in the department is a centralized hub of allied health professionals charged with coordinating transitions of care activities for complex post-discharge patients who might be at risk for readmission. This multidisciplinary team of case managers, a CHW, and a medical social worker, collaborate to facilitate follow-up care and support, access to primary and specialty care, and self-management education for patients and caregivers. The department works closely with multidisciplinary teams at other clinics in the academic health university’s clinical practice as shown in Figure 1. Since a gap in curriculum tailored for CHWs surfaced, the clinical and academic partners identified an opportunity for training expansion. The partners used a quality improvement approach to intervention delivery; leveraging the clinical partner’s existing readmission-prevention efforts to develop a structured Teach Back training. The goal of the training was to provide CHWs with didactic information and skill building practice focused on the integration of Teach Back into patient interactions, care coordination, and follow-up support. This article describes the development, implementation, and evaluation of a pilot Teach Back training curriculum for CHWs serving patients at high risk for readmission.

## Method

### Curriculum development

This project was reviewed by the Institutional Committee for the Protection of Human Subjects and the Quality Improvement Committee and determined to be quality improvement. Informed consent was not required for training participation for CHWs. The project exploration of the literature included a computerized search of the Academic Search Complete, MEDLINE, JSTOR, and PubMed. Search restrictions included English language only. Keywords included were Teach Back, CHW, workshop, health literacy workshop, nursing, resident, and physician. Various combinations of the keywords were used which resulted in 150 hits. These articles were screened for abstract relevance and 38 articles or curricula were finalized and sub-categorized into evidence of Teach Back use, how to Teach Back, and existing trainings and workshops as shown in Table 1. A majority of articles identified in the search cited curricula for healthcare providers from the Agency for Healthcare Research and Quality (AHRQ) Health Literacy Universal Precautions Toolkit, Always Use Teach Back! Toolkit, and North Carolina Program for Health Literacy, making these programs the largest resource for tailoring (35–39).

**TABLE 1:** Identified articles and curricula on Teach Back.

References	Title	Setting	Population	Citation
DeWalt et al. (33)	Developing and testing the health literacy universal precautions toolkit	Clinical practice	All staff at a practice, including physicians, nurses, receptionists, and business staff	DeWalt, D. A., Broucksou, K. A., Hawk, V., Brach, C., Hink, A., Rudd, R., and Callahan, L. Developing and testing the health literacy universal precautions toolkit. *Nursing outlook*, *59*(2), 85–94.
Wilson et al. (43)	Using the teach-back method to increase maternal immunization literacy among low-income pregnant women in Jamaica: A pilot study	Health centers	Nurses	Wilson, F. L., Mayeta-Peart, A., Parada-Webster, L., and Nordstrom, C. Using the teach-back method to increase maternal immunization literacy among low-income pregnant women in Jamaica: A pilot study. *Journal of Pediatric Nursing*, *27*(5), 451–459.
Street and De Haes (44)	Designing a curriculum for communication skills training from a theory and evidence-based perspective	Clinics	Clinicians	Street Jr, R. L., and De Haes, H. C. Designing a curriculum for communication skills training from a theory and evidence-based perspective. *Patient education and counseling*, *93*(1), 27–33.
Green et al. (32)	Addressing health literacy through clear health communication: a training program for internal medicine residents	Ambulatory clinic	Internal medicine residents	Green, J. A., Gonzaga, A. M., Cohen, E. D., and Spagnoletti, C. L. Addressing health literacy through clear health communication: a training program for internal medicine residents. *Patient education and counseling*, *95*(1), 76–82.
Lamiani and Furey (34)	Teaching nurses how to teach: An evaluation of a workshop on patient education	Academic hospital	Nurses	Lamiani, G., and Furey, A. Teaching nurses how to teach: An evaluation of a workshop on patient education. *Patient education and counseling*, *75*(2), 270–273.
Wittenberg et al. (45)	COMFORT SM communication for oncology nurses: Program overview and preliminary evaluation of a nationwide train-the-trainer course	Cancer centers	Oncology nurses	Wittenberg, E., Ferrell, B., Goldsmith, J., Ragan, S. L., and Buller, H. COMFORT SM communication for oncology nurses: Program overview and preliminary evaluation of a nationwide train-the-trainer course. *Patient education and counseling*, *101*(3), 467–474.
Smith et al. (46)	Evidence-based guidelines for teaching patient-centered interviewing	Clinics	Residents, physicians, nurse practitioners, and physician assistants	Smith, R. C., Marshall-Dorsey, A. A., Osborn, G. G., Shebroe, V., Lyles, J. S., Stoffelmayr, B. E., and Gardiner, J. C. Evidence-based guidelines for teaching patient-centered interviewing. *Patient Education and Counseling*, *39*(1), 27–36.
Morony et al. (47)	Enhancing communication skills for telehealth: development and implementation of a Teach-Back intervention for a national maternal and child health helpline in Australia	Telehealth	Nurses	Morony, S., Weir, K., Duncan, G., Biggs, J., Nutbeam, D., and Mccaffery, K. J. Enhancing communication skills for telehealth: development and implementation of a Teach-Back intervention for a national maternal and child health helpline in Australia. *BMC health services research*, *18*(1), 1–9.
Kripalani et al. (48)	Development and evaluation of a medication counseling workshop for physicians: can we improve on “take two pills and call me in the morning?”	Clinics	Internal medicine residents	Kripalani, S., Osborn, C. Y., Vaccarino, V., and Jacobson, T. A. Development and evaluation of a medication counseling workshop for physicians: can we improve on “take two pills and call me in the morning? “*Medical education online*, *16*(1), 7133.
Chandar et al. (49)	Assessing the link between modified “Teach Back” method and improvement in knowledge of the medical regimen among youth with kidney transplants: The application of digital media	Computer-based program	Patients	Chandar, J. J., Ludwig, D. A., Aguirre, J., Mattiazzi, A., Bielecka, M., Defreitas, M., and Delamater, A. M. Assessing the link between modified “Teach Back” method and improvement in knowledge of the medical regimen among youth with kidney transplants: The application of digital media. *Patient education and counseling*, *102*(5), 1035–1039.
Morony et al. (50)	Experiences of teach-back in a telephone health service	Maternal and child health helpline	Nurses	Morony, S., Weir, K., Duncan, G., Biggs, J., Nutbeam, D., and McCaffery, K. Experiences of teach-back in a telephone health service. *HLRP: Health Literacy Research and Practice*, *1*(4), e173–e181.
Yen and Leasure (35)	Use and effectiveness of the teach-back method in patient education and health outcomes	–	–	Yen, P. H., and Leasure, A. R. Use and effectiveness of the teach-back method in patient education and health outcomes. *Federal practitioner*, *36*(6), 284.
Berkman et al. (51)	Health literacy interventions and outcomes: an updated systematic review	–	–	Berkman, N. D., Sheridan, S. L., Donahue, K. E., Halpern, D. J., Viera, A., Crotty, K., and Viswanathan, M. Health literacy interventions and outcomes: an updated systematic review. *Evidence report/technology assessment*, (199), 1–941.
DeWalt et al. (52)	Health literacy universal precautions toolkit	–	–	DeWalt, D. A., Callahan, L. F., Hawk, V. H., Broucksou, K. A., Hink, A., Rudd, R., and Brach, C. Health literacy universal precautions toolkit. *Rockville, MD: Agency for Healthcare Research and Quality*, 1–227.
Anderson et al. (30)	The 5Ts for teach back: an operational definition for teach-back training	–	–	Anderson, K. M., Leister, S., and De Rego, R. The 5Ts for teach back: an operational definition for teach-back training. *HLRP: Health Literacy Research and Practice*, *4*(2), e94–e103.
Prochnow et al. (36)	Improving patient and caregiver new medication education using an innovative teach-back toolkit	Hospital	Nurses	Prochnow, J. A., Meiers, S. J., and Scheckel, M. M. Improving patient and caregiver new medication education using an innovative teach-back toolkit. *Journal of Nursing Care Quality*, *34*(2), 101–106.
Strosaker et al. (53)	Teaching residents to “teach-back”: does a structured curriculum including simulation improve pediatric resident communication skills?	Hospital	Pediatric residents	Strosaker, R. H., Kelly, S., Payne, W., Trapl, E., Boutry, M., and Scheid, A. Teaching residents to “teach-back”: does a structured curriculum including simulation improve pediatric resident communication skills? *Academic Pediatrics*, *12*(3), e13–e14.
Klingbeil and Gibson (54)	The teach back project: a system-wide evidence based practice implementation	Pediatric healthcare organization	multidisciplinary team members (including acute care, emergency room, and surgical nurses, dieticians, respiratory care practitioners, and occupational and physical therapists)	Klingbeil, C., and Gibson, C. The teach back project: a system-wide evidence based practice implementation. *Journal of Pediatric Nursing*, *42*, 81–85.
Joint Commission International (55)	Communicating clearly and effectively to patients: How to overcome common communication challenges in health care			Joint Commission International. Communicating clearly and effectively to patients: How to overcome common communication challenges in health care.
Morony et al. (29)	A stepped wedge cluster randomised trial of nurse-delivered Teach-Back in a consumer telehealth service	Maternal and child health helpline	Nurses	Morony, S., Weir, K. R., Bell, K. J., Biggs, J., Duncan, G., Nutbeam, D., and McCaffery, K. J. A stepped wedge cluster randomised trial of nurse-delivered Teach-Back in a consumer telehealth service. *PLoS One*, *13*(10), e0206473.
Talevski et al. (23)	Teach-back: A systematic review of implementation and impacts			Talevski, J., Wong Shee, A., Rasmussen, B., Kemp, G., and Beauchamp, A. Teach-back: A systematic review of implementation and impacts. *PLoS One*, *15*(4), e0231350.
Farris (56)	The teach back method			Farris, C. The Teach Back method. *Home healthcare now*, *33*(6), 344–345.
Wilson et al. (57)	Using the teach-back and Orem’s Self-care Deficit Nursing theory to increase childhood immunization communication among low-income mothers	Immunization clinic	Mothers	Wilson, F. L., Baker, L. M., Nordstrom, C. K., and Legwand, C. Using the teach-back and Orem’s Self-care Deficit Nursing theory to increase childhood immunization communication among low-income mothers. *Issues in comprehensive pediatric nursing*, *31*(1), 7–22.

The academic partner met weekly to review training curriculum material through an iterative process of brainstorming, content development, and improvement. Existing curricula for physicians and nurses were modified to make the content and clinical scenarios relevant to CHWs in a clinical setting. The curriculum was also informed by Adult Learning Principles to ensure the training was problem-oriented, relevant and impactful to the CHWs’ scope of work and to provide a rich learning experience (40). One team member drafted the initial curriculum sections and the remaining team members provided detailed feedback on content, relevancy, language, and aesthetics of the presentation. The lead member conducted a mock training session with the workgroup and the content delivery, timing, and presentation script were reviewed and refined. Each section of the curriculum and workshop went through several revisions until the workgroup reached a consensus on the final curriculum draft. To ensure clarity, accuracy and relevancy of content to the CHWs, the clinical leadership and CHW coordinators/managers (both were state certified CHWs) reviewed curriculum and provided feedback throughout development. The clinical leadership also contributed to article development.

### Curriculum implementation

A total of seven state certified CHWs from an outpatient clinical practice plan participated in the training sessions.^1^ CHW roles included scheduling and re-scheduling of appointments, following up about hospital discharge, working to increase adherence to vaccination schedules and mammography screening, referring patients to resources including food, transportation, housing, and assisting with paperwork and health insurance enrollment. The curriculum was delivered in three consecutive sessions (weeks) by WebEx video conference with team members from the academic partner as the instructors. The three training sessions are outlined in Table 2. Each training session was divided into a 1-h of didactic session followed by a 1-h session for discussion and skills practice.

**TABLE 2:** Teach Back training curriculum outline.

Session	Activity	Time (minutes)
**Week 1 training outline**		
Didactic session	Pre–training quiz (Table 3)	10 min
	Teach Back overview	30 min
	Plain language video^1^	5 min
	Plain language exercise	10 min
	Quiz on video	5 min
Practice and discussion session	Group activity with participants	40 min
	Discussion on group activity	10 min
	Post-training quiz (Table 3)	10 min
	Post-session survey (Table 5)	–
**Week 2 training outline**		
Didactic session	Previous week review	15 min
	Introduce teach-back tools/worksheets	30 min
	Video: What the Heck is Teach Back?^2^	5 min
	Discussion on video	10 min
Practice and discussion session	Discussion on submitted cases by participants	30 min
	Discussion on use of tools	30 min
	Post-session survey (Table 5)	–
**Week 3 training outline**		
Didactic session	Teach Back poll	10 min
	Revision of Teach Back	10 min
Practice and discussion session	Group activity: role play (using Table 4)	90 min
	Post-session survey (Table 5)	–

^1^https://www.youtube.com/watch?v=XiBZjpy3ibs.

^2^ https://www.youtube.com/watch?v=cllXBnHBiD4&feature=emb_logo.

#### Week 1 Teach Back introduction

In Week 1, the instructors reviewed health literacy concepts and discussed the importance of effectively communicating health information with patients. Teach Back was introduced as a communication skill to explain health information to patients and to assess if the patient can Teach Back the information and explain what they should do. The goal and elements of competence for Teach Back were presented alongside examples of verbal prompts, open-ended questions, and plain language. For the skills session, the CHWs were given a plain language activity to practice translating medical terminology into plain language. The CHWs were then given case scenarios to act out in groups and the instructors observed to provide feedback on how Teach Back was integrated into the case scenario.

#### Week 2 Teach Back didactics

After Week 1, CHWs were asked to submit a real-life case scenario example in which they used (or could use) Teach Back to help a patient think through their needs. In Week 2, the case scenarios were reviewed to assess how CHWs were using Teach Back. The discussion focused on possible barriers (e.g., time requirements during a patient visit or call) in using Teach Back and the responsibility of the CHW in providing clear communication in a clinical setting. In the practice session, CHWs observed a video demonstrating Teach Back and critiqued the scenario with the Teach Back elements of competence.

#### Week 3 Teach Back practice

Week 3 began with a review of Teach Back methodology and its importance. CHWs were then divided into two groups for a breakout session. A case scenario was provided to each CHW to act out and practice Teach Back in relevant clinical scenarios. The instructors observed and provided feedback to each group on their use and integration of Teach Back.

### Curriculum evaluation

A process evaluation was conducted to evaluate the overall training and the CHW’s understanding, skills, and use of Teach Back. First, in Week 1, a pre–post-training quiz was conducted to assess the identification of the correct use of Teach Back elements before and after the training session (Table 3). The quiz provided the instructors a baseline evaluation of the CHWs understanding of Teach Back. To complete Table 3, CHWs were given a case scenario to read which was followed by a set of eight true and false questions to assess inaccuracies in the use of Teach Back. A McNemar’s test was conducted to examine changes observed in the pre–post-training quiz responses. Second, in Week 3, CHWs were provided two case scenarios to practice Teach Back. Two groups took turns role playing as the CHW, patient, and observer. The instructors graded the participants on their correct use of eight Teach Back elements using a Criteria Checklist (Table 4). Third, in Week 3, a multiple-choice poll was administered with eight questions to gauge the Teach Back content comprehension and retention of CHWs. Some of the concerns voiced by the participants during previous two training sessions regarding use of Teach Back were included to quantify how many of the participants face the same challenges. Unfortunately, the poll data is not available for reporting due to WebEx deleting its stored content after 90 days of inception. Lastly, post-session surveys were administered after each of three sessions. The survey consisted of 10 questions to assess the quality of the training, instructors, changes in participants conviction, and confidence based on the Teach Back Conviction and Confidence Scale developed by the Institute for Healthcare Improvement (IHI) (Table 5) (41). The participants scored questions on a scale and a mean score was created. Since Week 1 was missing two participant responses, an unpaired *T*-test was performed on responses from Week 1 and Week 2. Responses from Week 2 and Week 3 were analyzed using a paired *T*-test and Wilcoxon signed-rank test.

**TABLE 3:** Teach Back pre–post-training quiz (Week 1).

Questions	True or false (T/F)
1. The community health worker (CHW) explained the steps and the procedure to the best of her understanding and ability.	T/F
2. The patient expressed understanding of the information.	T/F
3. The CHW was able to tell that the information she provided has been thoroughly understood by the patient.	T/F
4. The patient was very cooperative, understanding, and intelligent.	T/F
5. The CHW asked open-ended questions from the patient.	T/F
6. The CHW asked the patient to repeat in his own words what had just been explained to him.	T/F
7. The CHW used common words and plain language to explain what needed to be done.	T/F
8. The CHW used effective communication and Teach Back to help the patient understand what he needed to do.	T/F

**TABLE 4:** Teach Back criteria checklist (Week 3).

Criteria	Point (0 or 1)
1. Avoids using jargon and uses simple language.	
2. Asks open-ended questions.	
3. Gives plenty of time for patients to answer.	
4. Breaks down information into chunks for easier understanding.	
5. Asks to teach in their own words.	
6. Identifies any gaps in understanding.	
7. Fosters a shame free environment by choosing appropriate language.	
8. Repeats the teaching process until confirmed that the message has been understood.	
9. Total Score.	/8

**TABLE 5:** Teach Back post-session survey (Weeks 1–3).

Questions	
1. On a scale from 1 to 10, how convinced are you that it is important to use teach-back (ask patients to explain key information back in their own words)? (0 = Not at all important, 10 = Very important)	6. How would you rate the quality of the instructor? Not at all satisfied, slightly satisfied, moderately satisfied, very satisfied, completely satisfied
2. On a scale from 1 to 10, how confident are you in your ability to use teach-back (ask patients to explain key information back in their own words)? (0 = Not at all important, 10 = Very important)	7. Did you learn anything new? (with regards to the small group activity) Completely disagree, somewhat disagree, neutral, somewhat agree, completely agree
3. How often do you ask patients to explain back, in their own words, what they need to know or do to take care of themselves? I have been doing this for 6 months or more. I have been doing this for less than 6 months. I do not do it now, but plan to do this in the next month. I do not do it now, but plan to do this in the next 2–6 months. I do not do it now and do not plan to do this.	8. Was the course practical and/or easy to apply? Completely disagree, somewhat disagree, neutral, somewhat agree, completely agree
4. Did the training content meet your expectations? Completely disagree, somewhat disagree, neutral, somewhat agree, completely agree	9. Would you recommend the training to colleagues? Completely disagree, somewhat disagree, neutral, somewhat agree, completely agree
5. How would you rate the quality of the training? Not at all satisfied, slightly satisfied, moderately satisfied, very satisfied, completely satisfied	10. Do you have any suggestions to improve this course?

Questions 1–3 are based on the Conviction and Confidence Scale developed by the Institute for Healthcare Improvement (IHI) (41).

## Results

The McNemar’s test showed no statistically significant changes across all eight questions in the pre–post-training quiz after Week 1. However, trends can be identified from the results. For question 1 there was a 33% increase and for both questions 2 and 5 there was a 50% increase, in participants who went from a wrong quiz answer to correctly identifying the lack of Teach Back steps, patient understanding, and Teach Back skills in the case scenario. For question 8, 83% of the participants correctly identified that effective communication and Teach Back were not used. Yet, the small change in participants who had correct quiz answers for question 4 (0–35%) and question 7 (50–66%) indicated participants were unable to identify other inaccuracies in the case scenario. Based on Table 3, the highest score observed was 5/8 (63%) by one participant in terms of CHWs practicing Teach Back skills. The lowest score observed was 3/8 (38%) by three participants. Questions 2 and 8 had the lowest count observed (*n* = 1) and question 1 had the highest count (*n* = 6). The groups did not perform well on the fundamental elements of Teach Back observed in question 5 and 8 (i.e., asking the patient to explain in their own words, active listening, and repeating if the information had not been understood). Comparing across Weeks, no statistically significant changes in conviction or confidence mean scores were detected from the post-session survey. However, by the end of Week 3, 100% of the participants were highly convinced of the importance of using Teach Back with patients (mean score = ≥8). After Week 2, 57% of participants felt highly confident, 29% moderately confident (mean score = 5–7), and 14% felt not confident at all in their ability to use Teach Back (mean score = <5). After Week 3, 86% of participants felt highly confident and 14% felt moderately confident of their ability to use Teach Back (The ability to use Teach Back was not assessed after Week 1).

## Discussion

The Teach Back method has been identified by AHRQ and IHI as an effective method to increase patient health literacy (35). The effectiveness of Teach Back has been evaluated for a variety of patient and healthcare system outcomes, including patient satisfaction, knowledge, self-management, quality of life, 30-day readmissions, and health outcomes (21, 23–25, 27). Recent systematic reviews identified the use of Teach Back has positive impact on all of these, however, more high-quality randomized clinical trials are needed to further elucidate the effect of Teach Back (23, 35). Training for clinical staff in Teach Back is the commonly used approach to support implementation of both Teach Back and Transitional Care Management (TCM) interventions. Our pilot Teach Back training took place over three consecutive sessions (weeks) with contact times of 2 h each week. Overall, participants in the training self-reported the quality of the training and instructor as meeting their expectations (i.e., completely agree/highly satisfied). They also found the case scenarios used during the training were relevant and practical to their profession. They also agreed unanimously on having learned something new and would highly recommend the training to others. Similar to DeWalt et al. (33) participants noted that it would take some time to get comfortable with using Teach Back with patients.

Different from previous studies, these participants felt it would take more time to use Teach Back compared to their normal workflow (33). One suggestion from participants was to identify which patients would most benefit from application of Teach Back to minimize impact on their time and current metrics which were underlying the workflow concern. Concerns with workflow integration for Teach Back have been previously identified and best practice strategies to improve integration include the use of champions, team meetings, prompts, and audit and feedback (38). The training team met with leadership and discussed strategies they could apply, including adjustment or change in staff metrics, goal setting for Teach Back, providing tools to support use of Teach Back including checklists and observation tools, conducting observation of staff and methods for use in staff meetings including debrief and feedback support to encourage application. Similar to previously published studies, we found repeated exposure to the technique and providing time for staff practice improved confidence and conviction for using Teach Back from baseline (30). No statistically significant differences were observed in confidence and conviction across the three training sessions; however, small sample size limits the ability to detect differences (*n* = 7). In addition, the loss of data from our Week 1 post-assessment and Week 3 in training poll are limitations to our ability to assess impact. Future curriculum evaluations should be done with a larger audience to assess effect size.

The CHWs in our pilot training implementation came from a wide variety of clinical settings and provided a plethora of patient support roles. Like previous Teach Back training studies, we identified that flexibility and tailoring of our cases to the diverse roles of staff was helpful (30). Meeting with clinical leadership and CHW coordinators prior to our training to review and tailor case studies provided a useful substitute to on-site monitoring and process mapping of staff workflows and patient interactions, which would have been ideal. Unfortunately, the public health emergency due to COVID-19 prevented us from doing any on-site observation. Like previous Teach Back training studies, we identified staff who participated in our training were the least comfortable with asking open-ended questions and taking responsibility for patient understanding of instructions (30). During observation of the training by the academic partner, we noted a gap in staff’s use of motivational interviewing or active listening techniques. This mirrors our evaluation results which indicated this fundamental step of Teach Back had lower scores on the post-test for participants. Previous research has shown that elements of active listening such as asking open ended questions, reflecting and clarifying response are not common communication skills. These require deeper level, connective communication skills (42). Previous studies have similarly identified that Teach Back training is enhanced when coupled with specific communication skills training, particularly motivational interviewing or active listening (30). One limitation to our training due to the public health emergency is our lack of ability to observe the participants’ use of Teach Back skills post training. Ideally, we would have included structured observation of patient interactions to assess skill achievement and need for additional training. Future studies which embed both communication skills and Teach Back training together could enhance staff readiness and impact of the methods. To date Teach Back curricula have focused on clinical care staff including physicians and nurses. To our knowledge, the Teach Back training described here is one of the first applications of this method for CHWs. CHWs serve as a critical link between patient and healthcare providers, representing the patients’ communities and translating health information for populations with low literacy (16). Future studies applying and evaluating our training in this staff population would further the field.

## Data availability statement

The original contributions presented in this study are included in the article/Supplementary material, further inquiries can be directed to the corresponding author.

## Ethics statement

This study was reviewed by the Committee for the Protection of Human Subjects at The University of Texas Health Science Center at Houston (UTHealth) and deemed a quality improvement. Written informed consent for participation was not required for this study in accordance with the national legislation and the institutional requirements.

## Author contributions

GF, LT, and LH developed the training. JH led the manuscript development. All authors contributed to the manuscript writing and revisions, and approved the manuscript.

## Footnotes

1 See https://www.dshs.texas.gov/chw/CertRequire.aspx for more information on state certification.
