# Supplementary material for: Development, implementation, and evaluation of Teach Back curriculum for community health workers
Source: Front Med (Lausanne). 2022 Nov 3;9:918686. doi: 10.3389/fmed.2022.918686 (PMC9669070; doi:10.3389/fmed.2022.918686)
Supplement: Supplementary file 1 [file Table_1.DOCX]

| **Author (Year)** | **Title** | **Setting** | **Population** | **Citation** |
| --- | --- | --- | --- | --- |
| Anderson, K. (2020) | The 5Ts for teach back: an operational definition for teach-back training | - | - | Anderson, K. M., Leister, S., & De Rego, R. (2020). The 5Ts for teach back: an operational definition for teach-back training. HLRP: Health Literacy Research and Practice, 4(2), e94-e103. |
| Berkman, N. (2011) | Health literacy interventions and outcomes: an updated systematic review | - | - | Berkman, N. D., Sheridan, S. L., Donahue, K. E., Halpern, D. J., Viera, A., Crotty, K., ... & Viswanathan, M. (2011). Health literacy interventions and outcomes: an updated systematic review. Evidence report/technology assessment, (199), 1-941. |
| Chandar, J. (2019) | Assessing the link between modified ‘Teach Back’method and improvement in knowledge of the medical regimen among youth with kidney transplants: The application of digital media | Computer-based program | Patients | Chandar, J. J., Ludwig, D. A., Aguirre, J., Mattiazzi, A., Bielecka, M., Defreitas, M., & Delamater, A. M. (2019). Assessing the link between modified ‘Teach Back’method and improvement in knowledge of the medical regimen among youth with kidney transplants: The application of digital media. Patient education and counseling, 102(5), 1035-1039. |
| DeWalt, D. (2010) | Health literacy universal precautions toolkit | - | - | DeWalt, D. A., Callahan, L. F., Hawk, V. H., Broucksou, K. A., Hink, A., Rudd, R., & Brach, C. (2010). Health literacy universal precautions toolkit. Rockville, MD: Agency for Healthcare Research and Quality, 1-227. |
| DeWalt, D. (2011) | Developing and testing the health literacy universal precautions toolkit | Clinical practice | All staff at a practice, including physicians, nurses, receptionists, and business staff | DeWalt, D. A., Broucksou, K. A., Hawk, V., Brach, C., Hink, A., Rudd, R., & Callahan, L. (2011). Developing and testing the health literacy universal precautions toolkit. Nursing outlook, 59(2), 85-94. |
| Farris, C. (2015). | The teach back method |  |  | Farris, C. (2015). The teach back method. Home healthcare now, 33(6), 344-345. |
| Green, J. (2014) | Addressing health literacy through clear health communication: a training program for internal medicine residents | Ambulatory clinic | Internal medicine residents | Green, J. A., Gonzaga, A. M., Cohen, E. D., & Spagnoletti, C. L. (2014). Addressing health literacy through clear health communication: a training program for internal medicine residents. Patient education and counseling, 95(1), 76-82. |
| Joint Commission International. (2018) | Communicating clearly and effectively to patients: How to overcome common communication challenges in health care |  |  | Joint Commission International. (2018). Communicating clearly and effectively to patients: How to overcome common communication challenges in health care. |
| Klingbeil, C. (2018) | The teach back project: a system-wide evidence based practice implementation | Pediatric Healthcare organization | multidisciplinary team members (including acute care, emergency room, and surgical nurses, dieticians, respiratory care practitioners and occupational and physical therapists) | Klingbeil, C., & Gibson, C. (2018). The teach back project: a system-wide evidence based practice implementation. Journal of Pediatric Nursing, 42, 81-85. |
| Kripalani, S. (2011) | Development and evaluation of a medication counseling workshop for physicians: can we improve on ‘take two pills and call me in the morning’? | Clinics | Internal medicine residents | Kripalani, S., Osborn, C. Y., Vaccarino, V., & Jacobson, T. A. (2011). Development and evaluation of a medication counseling workshop for physicians: can we improve on ‘take two pills and call me in the morning’?. Medical education online, 16(1), 7133. |
| Lamiani, G. (2009) | Teaching nurses how to teach: An evaluation of a workshop on patient education | Academic hospital | Nurses | Lamiani, G., & Furey, A. (2009). Teaching nurses how to teach: An evaluation of a workshop on patient education. Patient education and counseling, 75(2), 270-273. |
| Morony, S. (2017) | Experiences of teach-back in a telephone health service | Maternal and child health helpline | Nurses | Morony, S., Weir, K., Duncan, G., Biggs, J., Nutbeam, D., & McCaffery, K. (2017). Experiences of teach-back in a telephone health service. HLRP: Health Literacy Research and Practice, 1(4), e173-e181. |
| Morony, S. (2018) | Enhancing communication skills for telehealth: development and implementation of a Teach-Back intervention for a national maternal and child health helpline in Australia | Telehealth | Nurses | Morony, S., Weir, K., Duncan, G., Biggs, J., Nutbeam, D., & Mccaffery, K. J. (2018). Enhancing communication skills for telehealth: development and implementation of a Teach-Back intervention for a national maternal and child health helpline in Australia. BMC health services research, 18(1), 1-9. |
| Morony, S. (2018) | A stepped wedge cluster randomised trial of nurse-delivered Teach-Back in a consumer telehealth service | Maternal and child health helpline | Nurses | Morony, S., Weir, K. R., Bell, K. J., Biggs, J., Duncan, G., Nutbeam, D., & McCaffery, K. J. (2018). A stepped wedge cluster randomised trial of nurse-delivered Teach-Back in a consumer telehealth service. PLoS One, 13(10), e0206473. |
| Prochnow, J. (2019) | Improving patient and caregiver new medication education using an innovative teach-back toolkit | Hospital | Nurses | Prochnow, J. A., Meiers, S. J., & Scheckel, M. M. (2019). Improving patient and caregiver new medication education using an innovative teach-back toolkit. Journal of Nursing Care Quality, 34(2), 101-106. |
| Smith, R. (2000) | Evidence-based guidelines for teaching patient-centered interviewing | Clinics | Residents, physicians, nurse practitioners, and physician assistants | Smith, R. C., Marshall-Dorsey, A. A., Osborn, G. G., Shebroe, V., Lyles, J. S., Stoffelmayr, B. E., ... & Gardiner, J. C. (2000). Evidence-based guidelines for teaching patient-centered interviewing. Patient Education and Counseling, 39(1), 27-36. |
| Street Jr, R. (2013) | Designing a curriculum for communication skills training from a theory and evidence-based perspective | Clinics | Clinicians | Street Jr, R. L., & De Haes, H. C. (2013). Designing a curriculum for communication skills training from a theory and evidence-based perspective. Patient education and counseling, 93(1), 27-33. |
| Strosaker, R. (2012) | Teaching residents to “teach-back”: does a structured curriculum including simulation improve pediatric resident communication skills? | Hospital | Pediatric residents | Strosaker, R. H., Kelly, S., Payne, W., Trapl, E., Boutry, M., & Scheid, A. (2012). Teaching residents to “teach-back”: does a structured curriculum including simulation improve pediatric resident communication skills?. Academic Pediatrics, 12(3), e13-e14. |
| Talevski, J., (2020) | Teach-back: A systematic review of implementation and impacts |  |  | Talevski, J., Wong Shee, A., Rasmussen, B., Kemp, G., & Beauchamp, A. (2020). Teach-back: A systematic review of implementation and impacts. PLoS One, 15(4), e0231350. |
| Wilson, F. (2008) | Using the teach-back and Orem's Self-care Deficit Nursing theory to increase childhood immunization communication among low-income mothers | Immunization clinic | Mothers | Wilson, F. L., Baker, L. M., Nordstrom, C. K., & Legwand, C. (2008). Using the teach-back and Orem's Self-care Deficit Nursing theory to increase childhood immunization communication among low-income mothers. Issues in comprehensive pediatric nursing, 31(1), 7-22. |
| Wilson, F. (2012) | Using the teach-back method to increase maternal immunization literacy among low-income pregnant women in Jamaica: A pilot study | Health Centers | Nurses | Wilson, F. L., Mayeta-Peart, A., Parada-Webster, L., & Nordstrom, C. (2012). Using the teach-back method to increase maternal immunization literacy among low-income pregnant women in Jamaica: A pilot study. Journal of Pediatric Nursing, 27(5), 451-459. |
| Wittenberg, E. (2008) | COMFORT™ SM communication for oncology nurses: Program overview and preliminary evaluation of a nationwide train-the-trainer course | Cancer centers | Oncology nurses | Wittenberg, E., Ferrell, B., Goldsmith, J., Ragan, S. L., & Buller, H. (2018). COMFORT™ SM communication for oncology nurses: Program overview and preliminary evaluation of a nationwide train-the-trainer course. Patient education and counseling, 101(3), 467-474. |
| Yen, P. (2019) | Use and effectiveness of the teach-back method in patient education and health outcomes | - | - | Yen, P. H., & Leasure, A. R. (2019). Use and effectiveness of the teach-back method in patient education and health outcomes. Federal practitioner, 36(6), 284. |
